# Methotrexate use in juvenile idiopathic arthritis and its impact on growth plate development: A prospective observational cohort study

**DOI:** 10.6026/973206300212680

**Published:** 2025-08-31

**Authors:** Shreyansh Ahirwar, Prashant Panale, Chetan Shokeen, Nitin Pratap Shishodia, Puneet Kamra, Sujit Kumar Singh, Mrudul Kyada

**Affiliations:** 1 Department of Dentistry, District Hospital, Vidisha, Madhya Pradesh, India; 2 Department of Pediatrics, Military Hospital, Bareilly, Uttar Pradesh, India; 3 Department of Pharmacology, Karnataka Medical College and Research Institute, Hubballi, Karnataka, India; 4 Department of Oral Medicine and Radiology, Seema Dental College and Hospital, Rishikesh, Uttarakhand, India; 5 Department of Orthopedics, SGT Medical College and Research Institute, Gurugram, Haryana, India; 6 Department of Orthopedics, ASJSATDS Medical College, Fatehpur, Uttar Pradesh, India

**Keywords:** Juvenile idiopathic arthritis, methotrexate, growth plate, growth velocity, IGF-1, MRI, pediatric rheumatology

## Abstract

Juvenile Idiopathic Arthritis (JIA) is a chronic inflammatory disease that can disrupt normal skeletal growth in children. Methotrexate (MTX) is widely used as a disease-modifying therapy in JIA, has uncertain effects on the developing growth plate and bone elongation. Hence, we enrolled 40 children with JIA, dividing them equally into MTX-exposed and non-MTX groups and evaluated growth plate morphology (via imaging), growth velocity (using height-for-age Z-scores) and biochemical markers (IGF-1, ALP, Vitamin D) over 12 months. The MTX group showed significantly reduced growth velocity (ΔZ = 0.31 vs. 0.58, p < 0.01), more epiphyseal irregularities on MRI (35% vs. 5%) and lower IGF-1 and ALP levels compared to controls. Although no severe skeletal defects were found, these findings suggest MTX may subtly affect growth, highlighting the need for careful longitudinal monitoring in treated children.

## Background:

Juvenile idiopathic arthritis (JIA) is the most common chronic rheumatic condition in children, defined by persistent joint inflammation and systemic symptoms, which often begin before the age of 16 and last at least six weeks [1]. The chronic inflammatory process in JIA not only affects joint integrity but also interferes with normal growth, particularly during periods of rapid skeletal development [2]. Growth retardation is driven by elevated cytokines, prolonged disease activity, and long-term corticosteroid use, all of which disrupt normal endochondral ossification [3, 4]. Methotrexate (MTX), a folate antagonist, is commonly recognized as the first-line disease-modifying antirheumatic medication (DMARD) for most subtypes of JIA, notably polyarticular and systemic [5, 6]. By targeting proliferating immune cells, MTX helps control synovial inflammation, reduce joint damage and minimize dependence on corticosteroids [7]. Although MTX has proven efficacy and a relatively favorable safety profile, its effects on growth plate morphology and linear growth velocity remain incompletely understood [8]. Recent clinical and experimental studies suggest that MTX may interfere with chondrocyte proliferation and matrix synthesis, potentially affecting growth plate structure and function [9, 10]. However, pediatric data are limited, and results are often confounded by disease severity, nutrition, and glucocorticoid co-therapy. Although radiography and MRI can detect subtle physeal changes, their use in MTX-treated JIA children remains limited [11, 12]. Therefore, it is of interest to describe and evaluate the impact of methotrexate therapy on growth plate development and linear growth in children with JIA using a multimodal approach that incorporates radiographic/MRI assessment, anthropometric measures (height-for-age Z-scores) and biochemical markers such as IGF-1, alkaline phosphatase and vitamin D.

## Methods:

This prospective observational cohort study was undertaken between January 2022 and December 2024 in the Departments of Pediatric Rheumatology and Orthopaedics at a tertiary care teaching hospital. The study involved 40 children aged 3 to 16 years who were diagnosed with Juvenile Idiopathic Arthritis (JIA) according to the International League of Associations for Rheumatology (ILAR) criteria. The participants were stratified into two groups based on their exposure to methotrexate (MTX): Group A consisted of children receiving MTX therapy for at least six months, while Group B included age- and sex-matched JIA patients who had never been exposed to MTX. Patients who had received systemic corticosteroids for more than three months, biologic agents, growth hormone therapy, or those with underlying congenital bone dysplasias or endocrine disorders were excluded from the study. Children with poor compliance to follow-up visits were also excluded. All individuals had their baseline demographics, illness features and anthropometric measurements recorded. Height, weight and body mass index (BMI) were determined using established methodologies at the baseline, 6 months and 12 months. Growth velocity was measured using height-for-age Z-scores based on World Health Organization (WHO) growth guidelines. Tanner staging was used where appropriate to assess pubertal development.

Radiological assessments included X-rays or MRI of the dominant knee or distal femur at baseline and at 12 months. These were interpreted by a blinded musculoskeletal radiologist to evaluate growth plate morphology, thickness and the presence of irregularities or premature physeal closure. Bone age was assessed using the Greulich and Pyle method. Growth velocity was measured using height-for-age Z-scores based on World Health Organization (WHO) growth guidelines. Tanner staging was used where appropriate to assess pubertal development. The primary outcome of the study was the difference in growth plate morphology between MTX-exposed and non-exposed groups after 12 months. Secondary outcomes included changes in height-for-age Z-scores and serum biomarker levels over the study duration, as well as correlation of cumulative MTX dose with growth velocity. The data were analyzed using SPSS version 26. Continuous variables were reported as mean ± SD and evaluated with independent t-tests or Mann-Whitney U tests. Categorical data were examined using Chi-square and Fisher's exact tests. Multivariate regression took into account age, gender, disease duration and baseline activity. P-values < 0.05 were considered significant. The Institutional Ethics Committee authorized the study procedure and each participant's parents or guardians provided informed consent. Children older than seven years old provided their consent. All assessments were part of regular clinical care and no new radiological or pharmacological therapies were used for study purposes.

## Results:

The study comprised 40 children with juvenile idiopathic arthritis (JIA): 20 in the methotrexate-exposed group (Group A) and 20 in the non-methotrexate group (Group B). The mean enrollment age was 9.6±3.1 years in Group A and 9.3±2.8 years in Group B (p = 0.71). The two groups were comparable in terms of sex distribution, disease subtype and length of illness, baseline height and BMI (Table 1 - see PDF). At 12 months, Group A showed a statistically significant reduction in mean height-for-age Z-score compared to Group B (-0.59±0.42 vs. -0.21±0.35, p = 0.014). Growth velocity was also lower in the methotrexate group (3.1±0.9 cm/year) compared to controls (4.2±1.1 cm/year, p = 0.008). Serum IGF-1 levels were significantly reduced in Group A at both 6 and 12 months (p < 0.05), while ALP and Vitamin D levels showed no significant intergroup differences (Table 2 - see PDF). Radiological assessment revealed morphological changes in the growth plate-such as irregular physis, focal thinning and early signs of physeal closure-in 7 of 20 (35%) patients in Group A, compared to 1 of 20 (5%) in Group B (p = 0.019). These changes were predominantly seen in the distal femur or proximal tibia on MRI. A small but statistically significant inverse connection was seen between cumulative methotrexate dose and growth velocity (r = -0.41, p = 0.038). Multivariate regression analysis adjusting for age, disease duration and baseline height confirmed methotrexate exposure as an independent predictor of reduced growth velocity and abnormal growth plate morphology.

## Discussion:

Growth impairment in children with juvenile idiopathic arthritis (JIA) is a major concern. It is often linked to chronic inflammation and cytokine-driven bone suppression. Glucocorticoid therapy and disease-modifying antirheumatic drugs (DMARDs), especially methotrexate (MTX), can also contribute [1, 5 and 10]. Our results confirm this concern. Twelve months of MTX use reduced linear growth and slowed growth velocity. Radiographs showed growth plate changes. The fall in height Z-scores mirrors prior reports linking antimetabolites to chondrocyte suppression and physeal arrest [2, 13]. About 35% of MTX-treated children showed abnormal growth plate morphology on imaging, echoing prior MRI studies of physeal dysplasia in chronic inflammation [14, 15]. MTX-treated children had markedly lower IGF-1, a key growth mediator, likely due to systemic inflammation and MTX-induced hepatic suppression [16, 17]. While ALP and Vitamin D levels remained comparable across groups, the selective IGF-1 depression reinforces its sensitivity as an early marker of growth compromise in chronic disease states [18]. Our findings are clinically relevant. Despite MTX being a cornerstone in JIA management due to its immunomodulatory efficacy and tolerability [6, 9], its potential impact on skeletal development should not be overlooked.

Growth retardation, even when subclinical, may culminate in permanent short stature or skeletal asymmetry if not addressed timely [19]. These findings call for regular growth and endocrine monitoring in children on MTX; long-term studies like Becker *et al.* [20] reported blunted growth with delayed therapy, while the German BIKER registry showed catch-up growth when disease control was early and MTX doses moderate [21]. It is essential to differentiate the effects of disease activity from drug exposure. While uncontrolled inflammation independently suppresses growth via pro-inflammatory cytokines like TNF-α and IL-6 [2, 4], MTX may compound this effect through direct impairment of chondrocyte proliferation and matrix synthesis [22]. Our adjusted analysis suggests MTX is an independent risk factor for impaired growth; early MRI changes in weight-bearing physis may signal subclinical damage, and recent pediatric imaging studies support MRI surveillance when growth deceleration appears [23]. Nevertheless, the therapeutic benefits of MTX cannot be understated. It remains one of the most effective DMARDs in reducing flare frequency and long-term joint damage [3, 7]. Importantly, growth outcomes improve substantially when disease control is achieved early; MTX's effect on growth may differ based on disease severity, cumulative dose, nutritional state and concurrent glucocorticoid treatment [11, 17]. Our study has limitations such as a small sample size and single-center design, which may limit generalizability. MRI was not performed for all joints, possibly underestimating physeal changes. We lacked longitudinal data on puberty and bone age and unmeasured factors like nutrition or subclinical hypothyroidism could have influenced growth. Strengths include a prospective design, age-matched controls, and multimodal assessment using anthropometry, biomarkers, and imaging. To our knowledge, this is among the few prospective Indian cohort studies evaluating MTX-related growth plate changes in JIA.

## Conclusion:

Methotrexate (MTX), despite its efficacy in managing Juvenile Idiopathic Arthritis (JIA), may subtly impact growth plate morphology and suppresses growth velocity over a 12-month period. MTX-exposed children exhibited mild epiphyseal changes on imaging, reduced height-for-age Z-score progression and lower serum IGF-1 and ALP levels, suggesting a possible biochemical basis for impaired growth, albeit within normal clinical limits. Thus, we show the importance of close growth monitoring in MTX-treated JIA patients and call for larger, longitudinal studies to better understands its long-term effects on skeletal development.
